# Knowledge, attitudes and medical practice regarding hepatitis B prevention and management among healthcare workers in Northern Vietnam

**DOI:** 10.1371/journal.pone.0223733

**Published:** 2019-10-14

**Authors:** Thi T. Hang Pham, Thuy X. Le, Dong T. Nguyen, Chau M. Luu, Bac D. Truong, Phu D. Tran, Mehlika Toy, Selen Bozkurt, Samuel So

**Affiliations:** 1 Asian Liver Center, Department of Surgery, Stanford University School of Medicine, Palo Alto, California, United States of America; 2 General Department of Preventive Medicine, Ministry of Health, Hanoi, Vietnam; 3 Department of Biomedical Informatics, Stanford University School of Medicine, Palo Alto, California, United States of America; Duke University, UNITED STATES

## Abstract

**Background and aim:**

Vietnam’s burden of liver cancer is largely due to its high prevalence of chronic hepatitis B virus (HBV) infection. This study aimed to examine healthcare workers’ (HCWs) knowledge, attitude and practices regarding HBV prevention and management.

**Methods:**

A cross-sectional survey among health care workers working at primary and tertiary facilities in two Northern provinces in Vietnam in 2017. A standardized questionnaire was administered to randomly selected HCWs. Multivariate regression was used to identify predictors of the HBV knowledge score.

**Results:**

Among the 314 participants, 75.5% did not know HBV infection at birth carries the highest risk of developing chronic infection. The median knowledge score was 25 out of 42 (59.5%). About one third (30.2%) wrongly believed that HBV can be transmitted through eating or sharing food with chronic hepatitis B patients. About 38.8% did not feel confident that the hepatitis B vaccine is safe. Only 30.1% provided correct answers to all the questions on injection safety. Up to 48.2% reported they consistently recap needles with two hands after injection, a practice that would put them at greater risk of needle stick injury. About 24.2% reported having been pricked by a needle at work within the past 12 months. More than 40% were concerned about having casual contact or sharing food with a person with chronic hepatitis B infection (CHB). In multivariate analysis, physicians scored significantly higher compared to other healthcare professionals. Having received training regarding hepatitis B within the last two years was also significantly associated with a better HBV knowledge score.

**Conclusions:**

Findings from the survey indicated an immediate need to implement an effective hepatitis B education and training program to build capacity among Vietnam’s healthcare workers in hepatitis B prevention and control and to dispel hepatitis B stigma.

## Introduction

Hepatitis B virus (HBV) is a serious global public health problem that can cause chronic infection and puts people at risk for premature death from cirrhosis and liver cancer. There is an estimated 257 million people living with chronic hepatitis B infection (CHB) in the world [1]. Asia and sub-Saharan Africa bear most of the burden of CHB, accounting for more than three quarters of the people with CHB in the world [2]. According to the 2016 Global Burden of Disease’s study, chronic HBV infection accounts for about 42% of all liver cancer deaths [3].

Vietnam has one of the highest prevalence of CHB. Hepatitis B surface antigen (HBsAg) prevalence were reported to be as high as 15–20% in the general population [4–7] with an estimated 9.6 million people living with CHB [8]. Each year an estimated 23,300 people in Vietnam die from hepatitis B related complications [9] and HBV- related liver cancer cases is projected to reach 58,600 by 2025, a 60% increase from 2005 [9]. Despite the inclusion of the hepatitis B vaccine in the expanded national infant immunization program since 2002, HBV continues to be a leading cause of mortality in Vietnam. Liver cancer ranked the third leading cause of cancer death in Vietnam in 2018, only after lung and stomach cancer [10]. Vietnam also ranks 5th in liver cancer mortality rate in the world [11]. Mathematical models predicted that Vietnam would continue to face an enormous burden of HBV-related liver disease in the future [9]. As perinatal transmission is the main cause of CHB in Vietnam, improving newborn hepatitis B vaccination coverage, particularly the birth dose given within 24 hours after delivery, is critical to eliminate mother-to-child transmission.

In 2015, Vietnam approved the first comprehensive national action plan for prevention and control of viral hepatitis that calls for concerted actions, including vaccination, prevention, testing and treatment of CHB [12]. In this plan, building the capacity of healthcare workers (HCWs) was identified as one of the pillars in the national strategies to prevent new HBV infection and reduce the complications of CHB. This study sought to assess the knowledge, attitudes, and medical practices of HCWs regarding HBV prevention and management. The findings from this survey will help to inform targeted training programs to build capacity of HCW to eliminate new HBV infections and reduce the burden of CHB and liver cancer.

## Methods

### Study population

This was a cross sectional study conducted from February through August 2017. Two provinces were selected from the list of 25 Northern provinces, representing the Northern Midlands and Mountains (Hoa Binh) and the Red River Delta (Quang Ninh). Fifteen public health clinics including two provincial hospitals, five district health clinics and eight commune health centers were selected for this study. All health care workers who provided health care to patients were eligible. The Scientific Research Committee of The Vietnam Family Planning Association reviewed and approved the study’s protocol and ethical clearance (reference number: 052017/HD5). Eligible HCWs were provided oral information about the study’s background, purposes and procedure. Only those who gave a written consent were included in the study.

The sample size was calculated using a single population proportion formula considering the following assumptions: the prevalence of HCWs who have correct knowledge and practice about hepatitis B prevention and management is 50%, a confidence interval of 95%, and a margin of error of 5%. The sample size was calculated to be 380. Because the total number of health care workers working in participating facilities is less than 10,000, we used correction formula and factored in an assumed non-response rate of 10%. The final sample size was 322. Number of study participants recruited at each clinic was proportional to its number of HCWs. Study participants were selected randomly from the list of HCWs.

### Questionnaire

The questionnaire was developed in Vietnamese based on the Asian Liver Center at Stanford University’s prior experiences in HBV knowledge surveys in HCWs [13–15]. The questionnaire was pre-tested on seven volunteer HCWs to test the questions’ language, flow and comprehension. After the pre-test, a few wording modifications were made so as the questions were interpreted as intended. It took a approximately 25 minutes to complete each interview. There were 42 questions on HBV knowledge (questions 8–26, 29–31, 37–42, 44–57), three questions on HBV attitudes (questions 61–63), and five questions on medical practices (questions 27, 28, 32–34) (See S1 File).

The Vietnam General Department of Preventive Medicine recruited and trained ten research assistants who were not affiliated with the participating health clinics to administer the questionnaire. Completed questionnaires were kept in locked cabinets and only be accessible to the study investigators.

### Statistical methods

The STATA 12.0 statistical software was used for data analysis. Basic descriptive statistics were generated from variables in the dataset. Continuous data were expressed as mean ± standard deviation (SD) and median (min-max), while categorical variables were expressed as proportions (%). HBV knowledge score was calculated based on the sum of correct answers to the 42 knowledge-based questions. A correct response to each question was given one point. Incorrect or missing responses received no points. First, univariate linear regression was performed to measure the association between HBV knowledge score and demographic factors (i.e., age, gender, occupation, specialties, level of facilities, and year of experience), previous experience with HBV (immunization, being tested for hepatitis B and having encountered with CHB patients), and whether the HCW received training on HBV in the past two years. Only the variables with p value <0.25 in univariate analysis were included into multivariate analysis [16]. Regression coefficients and their 95% confidence intervals (CIs) were used to provide a further clue regarding the relative importance of each independent variable on the outcome variable. Degree of statistical significance was declared at a p value ≤ 0.05.

## Results

### Demographics and HBV related experience of survey participants

Among the 322 HCWs that were approached, 314 agreed to participate in the study, giving a response rate of 97.5%. Characteristics of the study population (N = 314) are presented in Table 1. The ratio of male to female participants was 34:66 and 68.8% were between 25–45 years old. Majority of the HCWs (74.5%) have encountered CHB patients in the past. 81.2% reported they themselves had been tested for hepatitis B and 68.8% have received the hepatitis B vaccine. Nearly half of the participants (45.9%) reported having received training on HBV prevention, diagnosis or management within the last two years.

**Table 1:** Distribution of demographic factors and HBV related experience (N = 314).

	N	%
**Age**		
Under 25 years	54	17.2
25–34 years	152	48.4
35–45 years	64	20.4
Above 45 years	44	14.0
**Gender**		
Male	108	34.4
Female	206	65.6
**Occupation**		
Physician	99	31.5
Physician assistant	89	28.3
Nurse	50	15.9
Midwife	62	19.6
Others (medical student and lab technician)	14	4.4
**Work Department**		
Internal medicine	49	15.6
Obstetric	64	20.4
Pediatric	47	15.0
General medicine	64	20.4
Other	90	28.7
**Place of work**		
Commune health center	79	25.2
District health hospital	142	45.2
Province level hospital	93	29.6
**Years of experience**		
<5 years	99	31.5
5–10 years	112	35.7
>10 years	103	32.8
**Were you tested for HBV before?**		
Yes	255	81.2
No	59	18.8
**Did you receive the hepatitis B vaccine?**		
Yes	216	68.8
No	98	31.2
**Have you encountered any patient with chronic hepatitis B infection?**		
Yes	234	74.5
No	80	25
**Did you attend any training on HBV prevention, diagnosis or management in the past 2 years?**		
Yes	144	45.9
No	170	54.1

### Knowledge about HBV risk, prevention, and management

A total of 42 knowledge-based questions were used to assess HBV knowledge categories including prevalence, health risks, transmission, prevention, diagnosis, management and treatment as well as injection safety (Table 2). Majority of the participants (74.6%) were aware that CHB can cause serious complications such as liver cirrhosis, liver failure, liver cancer or premature death. About 41.4% provided the correct answer to the estimated prevalence of CHB in Vietnam. Only 39.5% were aware that mother-to-child transmission at birth is the most common cause of CHB in Vietnam, and only 24.5% were aware that newborns who become infected are at the highest risk of developing CHB (Table 2).

**Table 2:** Distribution of correct responses to HBV risk, prevention, and management (N = 314).

Questions	Correct answers	
	N	%
**Prevalence, risk, and sequelae of infection**		
Q8. How many percent of Vietnam population has CHB?	130	41.4
Q9. How did most people who have CHB in Vietnam got infected	124	39.5
Q10. Which age group is most likely to develop CHB infection after the initial infection?	77	24.5
Q11. Consequences of CHB infection?	234	74.6
**Transmission routes**		
Q12. Hepatitis B can be transmitted through handshake	279	88.9
Q13. Hepatitis B can be transmitted through unprotected sex	268	85.4
Q14. Hepatitis B can be transmitted through blood transfusion	279	88.9
Q15. Hepatitis B can be transmitted through sneezing or coughing	257	81.9
Q16. Hepatitis B can be transmitted through from mother to her child at birth	283	90.1
Q17. Hepatitis B can be transmitted through from eating or sharing food/utensils with HBV infected patients	219	69.8
**Prevention measures**		
Q18. Clean and cook food thoroughly can prevent HBV transmission	159	50.6
Q19. Hepatitis B vaccination to persons with no immunity can prevent HBV transmission	286	91.1
Q20. Do not reuse or share needles/syringes can prevent HBV transmission	289	92.0
Q21. Avoid sharing food/utensils or eating with a person with CHB can prevent HBV transmission	175	55.7
Q22. Use a condom can prevent HBV transmission	287	91.4
**HBV immunization**		
Q23. Who should be vaccinated to prevent HBV infection?	231	73.6
Q24. When would you give a healthy and stable baby the first dose of the hepatitis B vaccine?	281	89.5
Q25. Do you think the hepatitis B vaccine is safe?	192	61.2
Q26. If a pregnant woman has CHB, what would you do to protect the newborn from becoming infected?	233	74.2
**Injection safety**		
Q29. Wash hands with soap or disinfectant after each clinical procedure can prevent needle stick injury	259	82.5
Q30. Recap needle with two hands after use and discard immediately in a sharp-proof container can prevent needle stick injury	159	50.6
Q31. Do not recap needle and discard immediately in a sharp-proof container can prevent needle stick injury	148	47.1
**HBV screening**		
Q37. Would pregnant women need HBV screening test even though they don’t have any hepatic symptoms?	271	86.3
Q38. Would people living with HIV need HBV screening test even though they don’t have any hepatic symptoms?	255	81.2
Q39. Would men who have sex with men (MSM) need HBV screening test even though they don’t have hepatic symptoms?	256	81.5
Q40. Would family member of CHB patients need HBV screening test even though they don’t have hepatic symptoms?	284	90.5
Q41. Which single test would you order to confirm a patient has CHB?	278	88.5
Q42. Which single test would you order to know if a patient has immunity to HBV?	116	36.9
**HBV monitoring**		
Q47. Do all patients with CHB need to be regularly monitored and screened for liver cancer?	43	13.7
Q49. Would you order alpha-fetoprotein (AFP) to screen for liver cancer?	175	55.7
Q50. Would you order alanine transaminase (ALT) to screen for liver cancer?	102	32.5
Q51. Would you order (AST) to screen for liver cancer?	101	32.2
Q52. Would you order abdominal ultrasound to screen for liver cancer?	88	28.0
Q53. Would you order carcinoembryonic antigen (CEA) to screen for liver cancer?	83	26.4
Q54. Would you order alpha-fetoprotein (AFP) for regular monitoring of liver damage?	81	25.8
Q55. Would you order alanine transaminase (ALT) for regular monitoring of liver damage?	147	46.8
Q56. Would you order aspartate aminotransferase (AST) for regular monitoring of liver damage?	63	20.1
Q57. Would you order abdominal ultrasound for regular monitoring of liver damage?	84	26.8
**Treatment**		
Q44. What is the symptom most patients with CHB present?	43	13.7
Q45. Is there a cure for CHB?	193	61.5
Q46. Do you think that every patient with CHB need to receive antiviral treatment?	64	20.4
Q48. Without proper monitoring and treatment, what is the chance a patient would die of complications of CHB?	69	22.0

Most of the participants were aware that HBV can be transmitted through mother-to-child at birth (90.1%), unprotected sex (85.4%), and blood transfusion (88.9%). However, almost a third (30.2%) wrongly believed that HBV can be transmitted through eating with or sharing utensils with HBV infected patients, and 18.1% believed that HBV can be transmitted through sneezing or coughing. Almost half of the participants (49.4%) wrongly believed that cleaning and cooking food thoroughly can prevent HBV transmission, and 44.3% believed avoiding sharing food and eating utensils or eating with person with CHB can prevent HBV transmission. While the majority of participants (91.1%) were aware that HBV transmission can be prevented by hepatitis B vaccination, only 73.6% provided correct answers to the questions who should be vaccinated against hepatitis B. Only 61.2% were confident that the hepatitis B vaccine is very safe (Table 2).

Knowledge about injection safety was assessed by three questions (Table 2). Only about half of the respondents (50.6%) knew they should not recap the needle with two hands to prevent needle stick injury, and they should dispose of the used needle and syringe into a sharp container immediately without recapping the needle (47.1%).

Few (13.7%) were aware that patients with CHB often have no symptoms, and only 22.0% knew without proper monitoring and treatment, CHB carries up to a 25% risk of death from liver cancer or liver disease. Majority of the participants (88.5%) knew the single test used to confirm a patient has CHB is the hepatitis B surface antigen (HBsAg) test. However, knowledge about the tests to order to diagnose whether a patient has immunity to HBV, to monitor for liver damage, and to screen for liver cancer was poor (correct responses ranged from 20.1% to 55.7%). Knowledge about hepatitis B treatment was also lacking. Only 61.5% of the HCWs surveyed were aware that there is no curative treatment for CHB. Many (70.1%) wrongly believed that every CHB patients need to receive antiviral treatment.

### Attitude and medical practices regarding HBV prevention

Among the 284 participants who reported they were involved in administering injection to patients, 61.3% consistently wore gloves when giving injections. 48.2% reported they consistently recapped the needle with two hands after injection, putting them at greater risk of needle stick injury. About a quarter (24.2%) reported having been pricked with a needle at work in the past 12 months. About 34.4% reported that there were hepatitis B vaccine stock-out or no vaccine available for administration to newborn at their clinic (Table 3).

**Table 3:** Attitude and medical practice regarding HBV prevention (N = 314).

	N	%
**Is the hepatitis B vaccine available at your clinic for administration to newborn?**		
Yes, always available	184	58.6
Yes, but stock-out occurs	62	19.7
Not available	46	14.7
Don’t know	22	7.0
**Do you wear gloves when administrating injection to patients?**		
Always	174	61.3
Sometimes	97	34.1
Never	13	4.6
I am not involved in giving injection to patients	30	
**How do you often handle the needles after giving injection to patients?**		
I often recap needle with two hands after injection	137	48.2
I often recap needle with one hand after injection	94	33.1
I often don’t recap needle after injection	53	18.7
I am not involved in giving injection to patients	30	
**Have you been pricked with a needle at work in the past 12 months?**		
Yes	76	24.2
No	208	66.2
I am not involved in giving injection to patients	30	9.6
**Are there sharp-proof containers at your clinic for disposing needles and sharp objects?**		
Always	259	82.5
At some places	45	14.3
Not available	10	3.2
**Would you have any concern having casual contact or working together with a patient with CHB in the same office?**		
Yes	85	27.0
Somewhat concern	47	15.0
No	182	58.0
**Would you have any concern eating with (sharing food or utensils) with a patient with CHB?**		
Yes	70	22.3
Somewhat concern	58	18.5
No	186	59.2
**Would you have any concern if you child is in the same class with a child with CHB?**		
Yes	65	20.7
Somewhat concern	69	22.0
No	180	57.3

Among the HCWs surveyed, 42% expressed concerns with casual contact or working in the same place and 40.8% expressed concerns in sharing food with a patient with CHB. Similarly, 42.7% of the participants were either concerned or somewhat concerned if their children is in the same class with another student with CHB.

### HBV knowledge scores and associated factors

Out of a total score of 42, the average HBV knowledge score was 25.0 ± 4.5 (mean ± SD), median score was 25 (range 9–34, interquartile range (IQR) 23–28). Knowledge scores for the eight knowledge categories are presented in Table 4. Among the eight knowledge categories, median scores were lowest for CHB monitoring and treatment.

**Table 4:** Average and maximum score of different domains of HBV knowledge (N = 314).

	Average score ± SD	Median score (min-max)	Highest possible score
HBV prevalence and risk	1.8 ± 1.0	2 **(0–4)**	4
HBV transmission route	5.0 ± 1.3	5 (0–6)	6
HBV prevention measures	3.8 ± 1.1	4 (0–5)	5
Hepatitis B immunization	3.0 ± 1.0	3 (0–4)	4
Injection safety	1.8 ± 0.7	2 (0–3)	3
Hepatitis B testing	4.6 ± 1.2	5 (0–6)	6
CHB monitoring	3.1 ± 2.2	3 (0–8)	10
CHB treatment	1.4 ± 0.8	1 (0–4)	4
Total HBV knowledge score	25 ± 4.5	25 (9–34)	42

In multivariate linear regression analysis, physicians scored significantly higher than physician assistants, nurses, midwives, medical students, and lab technicians. Not having received training on HBV prevention and management in the past two years was associated with a 1.1 point lower total HBV knowledge score (p<0.05). Age, gender, years of work experience, work department and type of health facility had no significant impact on the total HBV knowledge scores. Prior encounter with HBV infected patients, history of having been tested or vaccinated against HBV were not associated with a significant improvement in their total HBV knowledge score (Table 5).

**Table 5:** Analysis of factors associated with HBV total knowledge score (N = 314).

		Univariate linear regression analysis			Multivariate linear regression analysis		
	Variables	Coef.	t	P	Adjusted Coef.	95% CI	P
**Age**	Under 25 years	Ref	-	-	Ref	-	-
	25–34 years	1.7	2.5	0.02	0.1	-1.9 to 2.00	0.94
	35–45 years	1.6	2.0	0.05	-0.1	-2.5 to 2.4	0.94
	Above 45 years	1.19	1.3	0.19	-0.3	-2.9 to 2.3	0.80
**Gender**	**Male**	Ref	-	-	Ref	-	-
	Female	-0.2	-0.4	0.72			
**Occupation**	Physician	Ref	-	-	Ref	-	-
	Physician assistant	-2.6	-4.1	<0.001	-2.5	-3.8 to -1.1	<0.001
	Nurse	-4.4	-3.6	<0.001	-4.6	-7.5to -1.8	0.001
	Midwife	-2.9	-3.9	<0.001	-3.0	-4.5 to -1.5	<0.001
	Others	-3.3	-4.7	<0.001	-3.6	-5.1 to -2.1	<0.001
**Work Department**	Internal medicine	Ref	-	-	Ref	-	-
	Obstetric	-0.9	-1.0	0.31	0.2	-1.5 to 2.0	0.78
	Pediatric	-0.4	-0.4	0.68	-0.6	-2.4 to 1.2	0.51
	General medicine	-1.2	-1.4	0.16	-0.6	-2.3 to 1.1	0.48
	Others	-0.8	-1.0	0.34	-0.1	-1.7 to 1.4	0.86
**Place of work**	Commune health center	Ref	-	-	Ref	-	-
	District health hospital	0.8	1.2	0.23	0.4	-0.8 to 1.7	0.51
	Province level hospital	-0.0	-0.0	0.98	0.4	-1.0 to 1.9	0.56
**Years of work experience**	<5 years	Ref	-	-	Ref	-	-
	5–10 years	0.8	1.2	0.22	0.4	-1.0 to 1.7	0.60
	>10 years	0.3	0.4	0.70	-0.10	-1.9 to 1.8	0.95
**Were you tested for HBV before?**	Yes	Ref	-	-	Ref	-	-
	No	-1.2	-1.7	0.08	-1.0	-2.3 to 0.3	0.11
**Did you receive the hepatitis B vaccine before?**	Yes	Ref	-	-	Ref	-	-
	No	-1.0	-1.8	0.68			
**Have you encountered any patient with chronic hepatitis B infection?**	Yes	Ref	-	-	Ref	-	-
	No	-1.6	-2.7	0.006	-1.1	-2.3 to 0.6	0.062
**Did you attend any training on HBV prevention, diagnosis or management in the past 2 years?**	Yes	Ref	-	-	Ref	-	-
	No	-1.0	-2.0	0.05	-1.1	-2.1 to -0.1	0.026

## Discussion

HBV is a serious public health problem in Vietnam. Our survey shows that even among healthcare professionals, basic knowledge about HBV facts including transmission routes and prevention measures is lacking. Majority of HCWs surveyed were not aware of the high prevalence of chronic HBV in Vietnam, and the major cause of CHB in Vietnam is mother-to- child transmission. About 42.7% of the participants mistakenly believed that most of the people who have CHB were infected from sexual transmission. Only one-fourth (24.5%) of the participants were aware the risk of developing chronic infection is highest when infected at birth. About 30.2% of the participants wrongly believed that HBV can be transmitted through eating or sharing food with CHB patients. These results are in line with the findings from previous surveys at selected health clinics in Vietnam that reported gaps in knowledge among HCWs and medical students regarding hepatitis B transmission, the health risks associated with chronic HBV infection and diagnostic tests for hepatitis B [17, 18]. The lack of knowledge and misconceptions among HCWs about the ways hepatitis B is transmitted and the high risk of developing CHB when infected at birth can result in missed opportunities for HBV prevention and vaccination, and discrimination against those who have CHB.

Since perinatal transmission is the major cause of CHB in Vietnam, infant vaccination plays a critical role in preventing new CHB infection. With the majority of births (98%) being attended by trained HCWs in Vietnam [19], HCWs are the most trusted advisor and influencer of the hepatitis B vaccine birth dose decision. Our study shows that uncertainty about the safety of the hepatitis B vaccine among HCWs was apparent. Only 61.2% of participants were confident that the hepatitis B vaccine is very safe. Surprisingly, our recent study in the same region showed that pregnant women and mothers seemed less skeptical about the safety of the hepatitis B vaccine than the HCWs surveyed. According to that study, 72.9% of women felt it is safe to give their baby the hepatitis B vaccine within the first 24 hours after birth if the newborn is healthy and stable [20]. Research has shown that health care providers’ acceptance of vaccine is a complex behavior that can be influenced by various factors including their knowledge, attitudes and beliefs as well as broader organizational, political, cultural factors [21, 22]. Vaccine hesitancy among HCWs could perpetuate suboptimal performance of infant HBV vaccination program. Further research to better understand the underlying determinants of vaccine hesitancy among HCWs in Vietnam is needed to inform the design of effective responses.

Hepatitis B is also an important occupational hazard for HCWs [1]. HCWs have up to a four-fold increased risk of acquiring HBV infection [23, 24]. According to the World Health Organization (WHO), 5.9% of HCWs each year are exposed to blood-borne HBV infection corresponding to about 66,000 HBV infections among HCWs worldwide [25]. The risk of HBV transmission in health-care settings is highest in countries where the overall prevalence of hepatitis B surface antigen is > 8% [26]. Some studies have reported knowledge about HBV among HCWs is deficient; consequently, proper precautions against blood-borne infections is lacking [8]. According to WHO estimates, hepatitis B vaccination coverage among HCWs remains poor varying from 18% (Africa) to 77% (Australia and New Zealand) [25]. Among the HCWs surveyed, 68.8% reported they have received the hepatitis B vaccine. This is consistent with the result from a recent survey among medical students in Vietnam in which 68.7% reported having received the hepatitis B vaccine [27]. This study, however, did not ask whether the participants completed the three vaccine doses. Adherence to standard universal infection control precautions in patient care is recommended to protect HCWs from contracting HBV and other blood-borne infections and to prevent the spread of infection from patient to patient. Previous studies have reported low compliance with injection safety standards among HCWs in Vietnam, ranging from 6.0 to 22.6% [28, 29]. The main risk factor for contracting blood-borne diseases among HCWs is direct contact with infectious materials, especially infected blood or from needle stick injury by contaminated body fluids [30]. In this study, only 61.3% reported they always wear gloves when giving injections. Among the HCWs surveyed, 26.1% reported having been pricked with a needle at work in the past 12 months, which was lower than the 65% reported in an earlier study in selected hospitals in Vietnam in 2012 [31]. The safest way to dispose a used needle is immediately placing the needle in a sharps disposal container to reduce the risk of needle sticks, cuts and punctures. In this survey, we found only 46.8% of HCWs reported adherence to this standard practice. Half (48.2%) of the participants in this study reported they routinely recapped the used needles with two hands after injection, putting them at greater risk of needle stick injury, compared with 35.0% in a report from India [32] and 81% from Sudan [33]. The findings re-emphasize the need for improving injection safety knowledge and training to prevent needle stick injuries and the transmission of HBV and other blood-borne infections in the health care settings.

This study reveals serious lack of knowledge about testing and interpretation of hepatitis B test results, CHB symptoms, and monitoring and treatment of patients with CHB. The median knowledge scores regarding CHB monitoring and treatment were the lowest among the eight HBV knowledge categories. The percentages of participants who provided correct answers to all the questions on the recommended tests to monitor for liver damage and to screen for liver cancer were only 1.6% and 2.5% respectively. Only 13.7% of the HCWs surveyed were aware that most of the CHB patient are asymptomatic. Providing basic information on CHB symptoms, monitoring and management should be addressed in future HCWs’ training.

When analyzed by the type of HCWs surveyed, physician assistants, nurses and midwives scored significantly lower than physicians. This is in line with the findings from other studies in which the type of healthcare professional have a significant association with HBV knowledge scores [13, 34–36]. Surprisingly, in this study, HCWs who had previous exposure to CHB patients or vaccinated against hepatitis B did not score better than those who did not. This is in contrast to a recent study in China that reported HCWs who had been screened for HBV and vaccinated scored significantly higher [13]. In this study, participants who attended training on HBV prevention, diagnosis or management in the last two years scored significantly higher than those who did not. This underlines a need for improved and targeted viral hepatitis training programs for HCWs, particularly for physician assistants, nurses and midwives, to improve knowledge and practice related to HBV prevention, monitoring and management, and injection safety.

Stigma surrounding caring for patients with blood-borne diseases including CHB in the health care settings can cause psychological and social consequences on patients and pose significant challenges to prevention, testing, and treatment efforts. In a recent study in Vietnam, almost one third of the participating nurses indicated their unwillingness to provide care for patients infected with HBV or hepatitis C [37]. The same study pointed out that the level of willingness is associated with individual confidence in protecting themselves against infection and negative attitudes towards HBV and hepatitis C infected patients. In this study, about 40% of participants were concerned or somewhat concerned about casual contact or eating with a person with CHB, or if their children are in the same class with a classmate who has CHB. HCWs’ misconceptions about HBV transmission and attitudes towards persons with CHB may lead to similar beliefs by the patients they serve. A recent study of pregnant women and mothers in the same region also found 37.4% had concerns about working with or sharing food with CHB patients and 40.8% expressed concerns in having their children attend the same class as a child with CHB [20]. Improving training and knowledge of HCWs about hepatitis B transmission would be important to eliminate HCWs’ and their patients’ misconceptions that result in disease stigma and discrimination.

## Study limitations

This study also has certain caveats that need to be taken into account. First, the answers are self-reported hence could not be validated. In addition, this study was conducted in two Northern provinces of Vietnam with low hepatitis B birth dose vaccination coverage and the results may not be representative to other parts of the country.

## Conclusion

This study found significant gaps in the knowledge of HCWs across all aspects of HBV including the high risk of CHB associated with infection at birth, vaccine safety, CHB symptoms, monitoring and treatment. Adherence among HCWs to standard injection safety practices, particularly on how to handle needles and syringes after injection, is also deficient. This study also re-affirms significant stigma among HCWs toward chronic HBV patients. The findings from this survey indicates an immediate need for implementing an effective training program including the use of the recently developed Vietnamese online viral hepatitis training course [38] to build capacity among healthcare workers within the Vietnam health system to improve hepatitis B prevention and management practices and to eliminate hepatitis B stigma.

## Supporting information

S1 File Survey questionnaire. (DOCX)

S2 File Dataset. (DTA)
