# Supplementary material for: Knowledge, attitudes and medical practice regarding hepatitis B prevention and management among healthcare workers in Northern Vietnam
Source: PLoS One. 2019 Oct 14;14(10):e0223733. doi: 10.1371/journal.pone.0223733 (PMC6791544; doi:10.1371/journal.pone.0223733)
Supplement: S1 File — (DOCX) [file pone.0223733.s001.docx]

**HEALTH CARE PROVIDER SURVEY**

**Facility ID:** _________

**PROVIDER DEMOGRAPHICS**

1. **Current age (years**): _____________ **2. Gender:**  Male  Female
2. **Type of provider (*Please check ONE*):**

Physician  Physician Assistant  Nurse  Midwife

Others (specify)

1. **Specialty (*Please check ONE*):**

Internal Medicine  Pediatrics  General Medicine

Obstetrics  Other (specify): _____________

1. **Place of work (*Please check ONE*):**

Commune health center  District level hospital  Province level hospital _

1. **Years of working experience: _____________**
2. **Education level (*Please check ONE*):**

Two-year college  University  Master or above

**DISEASE BURDEN, TRANSMISION ROUTES AND PREVENTION MEASURES**

1. **In your opinion, approximately how many percent of Vietnam population has chronic hepatitis B? (*Please check ONE*):**
2. 1% (1 in 100)
3. 5% (1 in 20 person)
4. 15% (1 in 7 person)
5. 25% (1 in 4 person)

1. **In your opinion, how did most people who have chronic hepatitis B in Vietnam got infected? (*Please check ONE*):**
2. at birth from an infected mother
3. unsafe injections
4. contaminated food or water
5. unprotected sex

1. **A person is most likely to develop chronic hepatitis B infection after the initial infection at? (*Please check ONE*):**
2. Newborns
3. Teens
4. Middle-age and seniors
5. Age is not a factor

1. **In your opinion, chronic HBV can cause which of the followings? (*Please check ONE*):**
2. Liver cirrhosis
3. Liver failure
4. Liver cancer
5. Premature death
6. All of the above

**Question 12-18: How could HBV be transmitted?**

1. **Shaking hands with a person with chronic HBV**
2. True
3. False
4. Don’t know
5. **Having unprotected sex with a person with chronic HBV**
6. True
7. False
8. Don’t know
9. **Having blood transfusion**
10. True
11. False
12. Don’t know
13. **Sneezing or coughing**
14. True
15. False
16. Don’t know
17. **From mother to her child at birth**
18. True
19. False
20. Don’t know
21. **Eating with or sharing food and utensils with a person with chronic HBV**
22. True
23. False
24. Don’t know

**Questions 19-23: What can prevent hepatitis B transmission?**

1. **Clean and cook food thoroughly**
2. True
3. False
4. Don’t know
5. **Provide HBV vaccination to persons with no immunity**
6. True
7. False
8. Don’t know

1. **Do not reuse or share needles/syringes**
2. True
3. False
4. Don’t know
5. **Avoid sharing food and utensils or eating with a person with chronic HBV**
6. True
7. False
8. Don’t know
9. **Use condom**
10. True
11. False
12. Don’t know
13. **Who needs to be vaccinated to prevent hepatitis B infection? (*Please check ONE)***
14. All healthy and stable newborns
15. Family members of someone who has chronic HBV and have not been vaccinated
16. Sex partner of persons with chronic HBV and have not been vaccinated
17. Healthcare workers without immunity
18. All of the above
19. **When would you give a healthy and stable baby the first dose of HBV vaccine? (*Please check ONE*)**
20. Within the first 24 hours of birth
21. 1- 7 days old
22. 1 month old
23. **Do you think the hepatitis B vaccine is safe (*Please check ONE*):**
24. Very safe
25. Maybe safe
26. Not very safe
27. **If a pregnant woman has chronic hepatitis B, what would you do to protect the newborn from becoming infected? (Please check ONE)**
28. Administer hepatitis B vaccine to the pregnant woman
29. Administer the first dose of hepatitis B vaccine and the HBIG shot within 12 hours of birth then complete the vaccine series
30. Administer the first dose of hepatitis B vaccine and the HBIG shot after 48 hours of birth then complete the vaccine series
31. **Is HBV vaccine available at your clinic for administration to newborn? (*Please check ONE*):**
32. Yes, always available
33. Yes, but stock-out occurs
34. Not available
35. There is no delivery at the clinic
36. **Have you been stuck with a needle at work in the past 12 months**
37. Yes
38. No

**Question 29-31: As a healthcare worker, what would you do to prevent needlestick injury?**

1. **Wash hands with soap or disinfectant after each clinical procedure**
2. True
3. False
4. Don’t know
5. **Recap needle with two hands after use and discard immediately in a sharp-proof container**
6. True
7. False
8. Don’t know
9. **Do not recap needle and discard immediately in a sharp-proof container**
10. True
11. False
12. Don’t know
13. **Are there sharp-proof containers at your clinic for disposing needles and sharp objects? (*Please check ONE*):**
14. Always
15. At some places
16. Not available
17. **Do you wear glove when administrating injection to patients? (*Please check ONE*):**
18. Always
19. Sometimes
20. Never
21. **How do you often handle the needles after giving injection to patients? (Please check ONE):**
22. I often recap needle with two hands after injection
23. I often recap needle with one hand after injection
24. I often don’t recap needle after injection
25. I am not involved in giving injection to patients
26. **Have you been tested for HBV before?**
27. Yes
28. No-> go to Q36

34.1

1. **Have you been vaccinated against HBV?**
2. Yes
3. No

**DIAGNOSIS AND MANAGEMENT**

**Question 37-40: Would you order hepatitis B screening test for following patients who don’t have hepatic symptoms?**

1. **Pregnant women**
2. Yes
3. No

1. **Persons infected with HIV**
2. Yes
3. No
4. **Men who have sex with men (MSM)**
5. Yes
6. No
7. **Family members of chronic hepatitis B patients**
8. Yes
9. No
10. **Which single test would you order to confirm that a patient has chronic hepatitis B? (Please check ONE):**
11. HBsAg
12. Anti-HBs
13. Anti-HBc
14. HBeAg
15. **Which single test would you order to know if a patient has immunity to hepatitis B? (Please check ONE):**
16. HBsAg
17. Anti-HBs
18. Anti-HBc
19. Anti-HBe
20. HBeAg
21. **Have you encountered any patient with chronic hepatitis B infection?**
22. Yes
23. No
24. **In your opinion, what is the symptom most patients with chronic hepatitis B present? (Please check ONE):**
25. Headache and fatigue
26. Nausea or vomiting
27. Loss of appetite
28. Jaundice
29. All of the above
30. None, there are usually no symptoms
31. **Which of following statements is true about HBV treatment? (*Please check ONE*):**
32. HBV is curable
33. There is no cure, but there are medications effective to manage and control the disease
34. There is no treatment available, but there are herbal medicine that can help to slower the disease progression.
35. **Do you think that all patients with chronic HBV need to be treated? (*Please check ONE*):**
36. Yes, all patients with chronic HBV should receive treatment as soon as possible
37. Only patients with active liver damage or cirrhosis need to be treated
38. There is no need to treat HBV because there is no cure yet
39. **Which of the followings is correct about monitoring chronic HBV patients? (*Please check ONE*):**
40. Only patients with symptoms need to be regularly monitored and screened
41. Only patients who are on HBV treatment need to be regularly monitored and screened
42. All patients with chronic HBV need to be regularly monitored and screened, regardless of treatment indication
43. **Without proper monitoring and treatment, what is the chance a patients would die of complications of chronic hepatitis B? (*Please check ONE*):**
44. Less than 5%
45. 5-10%
46. 15-25%
47. >30-40%%
48. Over 40%

**Question 49-52: Which of the following test would you order to screen for liver cancer?**

1. **Alpha-fetoprotein (AFP)**
2. Yes
3. No
4. Don’t know
5. **Alanine transaminase (ALT)**
6. Yes
7. No
8. Don’t know
9. **Aspartate aminotransferase (AST)**
10. Yes
11. No
12. Don’t know
13. **Abdominal ultrasound**
14. Yes
15. No
16. Don’t know
17. **Carcinoembryonic antigen (CEA)**
18. Yes
19. No
20. Don’t know

**Question 53-56: Which of the following test would you order for regular monitoring of liver damage?**

1. **Alpha-fetoprotein (AFP)**
2. Yes
3. No
4. Don’t know
5. **Alanine transaminase (ALT)**
6. Yes
7. No
8. Don’t know
9. **Aspartate aminotransferase (AST)**
10. Yes
11. No
12. Don’t know
13. **Abdominal ultrasound**
14. Yes
15. No
16. Don’t know

**Question 57-59 Have you attended training on following topics in the past 2 years?**

1. **Prevention of Hepatitis B**
2. Yes
3. No
4. **Diagnosis of Hepatitis B**
5. Yes
6. No
7. **Management of patients with Hepatitis B**
8. Yes
9. No
10. **Would you have any concern having casual contact or working together with a chronic HBV patients in the same office?**
11. Yes
12. No
13. Somewhat concern
14. **Would you have any concern eating with (sharing food or utensils) with a chronic HVBV patient?**
15. Yes
16. No
17. Somewhat concern
18. **Would you have any concern if you child is in the same class with a chronic HVB kid?**
19. Yes
20. No
21. Somewhat concern
